# A National Evaluation of Intercostal Chest Drain Removal Strategies

**DOI:** 10.1016/j.chest.2025.10.027

**Published:** 2025-11-04

**Authors:** Niki Veale, Anthony W. Martinelli, Dheeraj Sethi, Phillip De Souza, Khaing Zar Mon, Joyce Oi Suet Cheng, David Morrow, May Sam, Irfan Saleem, Kay Por Yip, Jennifer Kerks, David Henshall, Tobias Smitherman-Cairns, Katherine Smith, Daniel Mitchell, Karl Jackson, Benjamin Pippard, Seemab Paul, Waheed Mohammad, James Hyman, Benjamin Rowlands, Samantha Bosence, Catharine Pearce, Ben Probyn, Richard Thorley, Matthew Mitchell, Andrew Griffiths, Richard Westley, Abdullah Bin Huda, Asim Mehmood, Abid Khan, Vern Tee, Rachel Crooks, Paul Minnis, Lewis Standing, Wei Hann Ong, M. Salman Rashid, Ahmed Salih, Eve Lynn Koh, Ching Khai Ho, Yiwen Soo, Matthew Hayes, Clodagh Holmes, Fatima Al-Arrayed, Abeer Saad, Beenish Iqbal, Sam Trewick, Patrick Goodley, Jonathan Oldershaw, Elizabeth Thompson, Alexandra Hodge, Mohamed Gadallah, Rahul Bhat, Eleanor Barton, Anand Sundaralingam, Osei Kankam, James Quinn, John P. Corcoran, Steven P. Walker, Avinash Aujayeb, Jurgen Herre, Akhilesh Jha, Stefan J. Marciniak, Najib M. Rahman, Rob J. Hallifax

**Affiliations:** a Cambridge University Hospitals NHS Foundation Trust, Cambridge, England; b Royal Papworth Hospital NHS Foundation Trust, Cambridge, England; c Cambridge Institute of Therapeutic Immunology & Infectious Disease, University of Cambridge, Cambridge, England; d Norfolk and Norwich University Hospitals NHS Foundation Trust, Norwich, England; e Mid and South Essex NHS Foundation Trust, Essex, England; f Institute of Inflammation and Ageing, University of Birmingham, Birmingham, England; g University Hospitals Birmingham NHS Foundation Trust, Birmingham, England; h Maidstone and Tunbridge Wells NHS Trust, Maidstone, England; i Deanery of Clinical Sciences, College of Medicine & Veterinary Medicine, The University of Edinburgh, Edinburgh, Scotland; j Newcastle upon Tyne Hospitals NHS Foundation Trust, Newcastle, England; k Northumbria Healthcare NHS Foundation Trust, Newcastle, England; l South Tyneside and Sunderland NHS Foundation Trust, Sunderland, England; m County Durham and Darlington NHS Foundation Trust, Darlington, England; n Royal Devon University Healthcare NHS Foundation Trust, Exeter, England; o University Hospitals Plymouth NHS Trust, Plymouth, England; p Aneurin Bevan University Health Board, Newport, Wales; q Sheffield Teaching Hospitals NHS Foundation Trust, Sheffield, England; r Mid Yorkshire Teaching NHS Trust, Wakefield, England; s Northern Health and Social Care Trust, Antrim, Northern Ireland; t North Tees and Hartlepool NHS Foundation Trust, Stockton-on-Tees, England; u Calderdale and Huddersfield NHS Foundation Trust, Huddersfield, England; v James Paget University Hospitals NHS Foundation Trust, Great Yarmouth, England; w North Cumbria Integrated Care NHS Foundation Trust, Carlisle, England; x North West Anglia NHS Foundation Trust, Huntingdon, England; y Oxford University Hospitals NHS Foundation Trust, Oxford, England; z Manchester University NHS Foundation Trust, Manchester, England; aa Royal Free London NHS Foundation Trust, London, England; bb United Lincolnshire Hospitals NHS Trust, Lincoln, England; cc George Eliot Hospital NHS Trust, Nuneaton, England; dd North Bristol NHS Trust, Bristol, England; ee East Sussex Healthcare NHS Trust, Sussex, England; ff Oxford Respiratory Trials Unit, University of Oxford, Oxford, England; gg Victor Phillip Dahdaleh Heart and Lung Research Institute, University of Cambridge, Cambridge, England; hh Oxford NIHR Biomedical Research Centre, Oxford, England; ii Chinese Academy of Medicine Oxford Institute, Oxford, England

**Keywords:** clamping, digital suction, pneumothorax, tension pneumothorax

## Abstract

**Background:**

Management of spontaneous pneumothorax often involves intercostal chest drain (ICD) insertion. Determining when to remove the ICD is controversial, with significant variation in practice. Establishing optimal ICD management in pneumothorax could reduce morbidity and improve cost-effectiveness.

**Research Question:**

Do ICD removal strategies, including clamping and use of digital air leak devices, impact the risk of pneumothorax recurrence, need for repeat pleural procedures, or length of stay?

**Study Design and Methods:**

We conducted a multicenter retrospective analysis of patients requiring ICD insertion for spontaneous pneumothorax from May 2021 to October 2023. Data were collected on demographics, clinical course, ICD removal strategy, pneumothorax recurrence (early and late), and repeat pleural intervention.

**Results:**

A total of 791 admissions from 27 centers were included. The 30-day recurrence of pneumothorax was 13.0% (n = 103). Clamping trials were undertaken in 32.6% of cases (n = 258), but recurrence of pneumothorax was not significantly different in clamped compared with nonclamped groups (14.0% vs 12.6%, respectively; *P* = .67). Clamping identified pleural air reaccumulation in 24 episodes (9.3% of the clamped group). Of 234 cases where clamping did not identify air leak, 35 patients (15.0%) developed recurrent pneumothorax. Of the 533 patients whose drains were not clamped, 67 (12.6% of the group) developed recurrence. The median length of stay was 6 (clamped) vs 5 days (nonclamped) (*P* = .08). Adverse events associated with clamping were few (n = 6), but included tension pneumothorax (n = 1). Digital air leak devices combined with clamping resulted in the lowest rates of pneumothorax recurrence; however, this approach was rare (n = 24, 0.0% recurrence within 7 days).

**Interpretation:**

Our results indicate that recurrent pneumothorax after ICD removal is a common complication. Clamping trials are safe but do not appear to be associated with reduced rates of recurrent pneumothorax. An ultracautious approach using digital air leak devices in combination with clamping could represent a viable strategy in selected patients.

## Graphical Abstract

**Figure:**
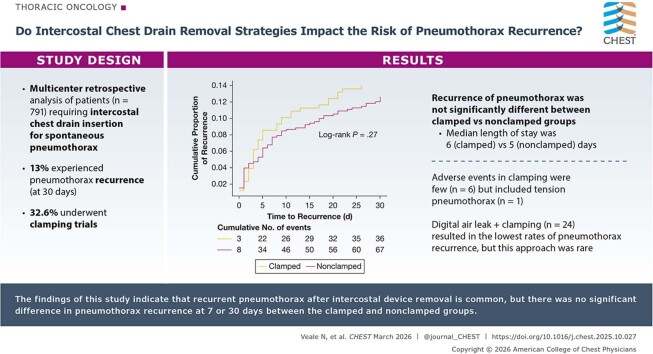

FOR EDITORIAL COMMENT, SEE PAGE 596

**Take-Home Points**

**Research** **Question:** Do intercostal chest drain removal strategies, including clamping, impact the risk of pneumothorax recurrence?

**Results:** There was no significant difference in pneumothorax recurrence at 7 or 30 days between the clamped and nonclamped groups.

**Interpretation:** Our results show that clamping trials are generally safe, but do not reduce the rate of recurrent pneumothorax or need for repeat pleural intervention.

Spontaneous pneumothorax remains a common driver for hospital attendance, with the UK hospitalization rate having returned to its prepandemic baseline of a mean 2.0 admissions per 100,000 population per month.^1^ Of these admissions, primary spontaneous pneumothorax (PSP), without evidence of underlying lung disease, is less common than secondary spontaneous pneumothorax (SSP), which is most frequently related to COPD-emphysema.^2^ Management of pneumothorax by insertion of an intercostal chest drain (ICD) (thoracostomy tube or chest tube) to allow evacuation of air remains the standard of care in SSP, cases of PSP with high-risk features (eg, hemodynamic instability), and cases of PSP refractory to conservative management or needle aspiration.^3^^,^^4^ Having been admitted with spontaneous pneumothorax and undergone ICD insertion, patients are committed to inpatient management until safe removal of the ICD, which can result in prolonged length of stay and significant cost to health care providers.^5^, ^6^, ^7^ Therefore, it is clear that optimizing all aspects of this pathway from emergency department presentation to discharge has the potential to drive benefits for both patients and clinicians.

Although decision-making regarding the need for ICD insertion has been and is the subject of a number of trials, the process for deciding when to remove the ICD, allowing discharge from hospital, has been less well studied.^8^^,^^9^ This decision point marks a critical juncture because removing an ICD while air leak is ongoing can result in recurrent pneumothorax, clinical deterioration, and repeat pleural intervention, thus prolonging inpatient stay, and discomfort and risk for the patient concerned. Recurrence of pneumothorax within 7 days of hospital discharge—a time point thought likely to represent failed closure of the original pleural defect rather than a second puncture—has been calculated as 4.1% (male) and 4.5% (female) for PSP, and 6.7% (male) and 5.6% (female) for SSP.^2^ Therefore, physicians use a variety of approaches to confirm resolution of air leak including the use of digital suction devices to quantify air flow, clamping the ICD to mimic removal, and clinical assessment via appraisal of visible air leak (bubbling) within the drainage bottle’s underwater seal. The efficacy of digital air leak devices has been best assessed in the postsurgical context, where their use has been shown to reduce duration of ICD insertion and length of stay, but this has not yet been extended to spontaneous pneumothorax.^10^ The use of clamping trials is largely based on anecdotal evidence and single-center retrospective studies, with the only prospective studies focused on air leak postsurgery or trauma.^11^, ^12^, ^13^, ^14^ Motivations for avoiding clamping trials include potentially prolonging discomfort, lack of utility, and the possibility of precipitating tension pneumothorax by blockage of air removal.^15^ Case reports of tension pneumothorax secondary to ICD clamping are, however, absent from the literature. Given the paucity of evidence, it is unsurprising that choice of ICD removal strategy is highly variable and that assessing clamping utility was identified as an open research question in the 2023 British Thoracic Society Clinical Statement on pleural procedures.^16^, ^17^, ^18^

The aim of this study was to assess whether differing ICD removal practices (clamped or not, and the use of digital suction) are associated with altered rates of recurrent pneumothorax, repeat pleural procedures, or length of stay.

## Study Design and Methods

The Characterising Leak of Air in Medical Pneumothorax (CLAMP) project was a multicenter retrospective observational study. Sites were recruited from across the United Kingdom via the Integrated Respiratory Research Collaborative (INSPIRE), a national respiratory resident-led collaborative research network.^19^ The study was approved by the lead site, Cambridge University Hospitals NHS Foundation Trust, as a multicenter service evaluation (No. 5574) with Caldicott Guardian and Information Governance approval. Additional local approval processes were undertaken within each center. Anonymized participant data were collected and managed using REDCap hosted at the University of Cambridge.^20^^,^^21^

A complete-case analysis was performed for patients admitted to hospital between May 2021 and October 2023 who underwent ICD insertion to manage PSP or SSP, with missing counts for key analytical variables reported (e-Table 1). For study inclusion, it was necessary that pneumothorax had persisted for > 24 hours based on clinical assessment and that it had improved such that ICD removal was being considered. Two or more admissions with pneumothorax from the same patient could be included as separate episodes if the second event was > 30 days from the index admission.

Exclusion criteria were < 16 years of age, traumatic pneumothorax, iatrogenic pneumothorax, postsurgical air leak, and bilateral pneumothorax. Patients who had chemical or autologous blood pleurodesis performed during the admission were also excluded. Patients who had persistent clinical air leak (ie, ongoing bubbling via underwater seal) and underwent surgery for pneumothorax without a clamping trial were not included. Neither patients who died with an ICD in situ nor patients where the ICD was removed inadvertently were included in the analysis.

Demographic, medical history, and clinical progress data were obtained retrospectively from paper and electronic medical records. Where the presenting pneumothorax was classed as SSP, the etiology of underlying lung disease was recorded. Patients > 50 years of age with a significant smoking history were classed as SSP. We collected information regarding use of thoracic suction, including whether a digital air leak devices was used. Because data were collected retrospectively, there were no fixed criteria determining whether a clamping trial was undertaken, with the decision having been made by the treating clinician. For patients where the ICD was not clamped, usual care included monitoring of air leak using an underwater seal. If a clamping trial was undertaken, radiologic findings after clamping and subsequent outcomes (ie, reaccumulation of pneumothorax, ICD removal) were recorded, specifically including any adverse events related to clamping.

End points were the following: diagnosed recurrence of pneumothorax within 7 days (early recurrence, most likely representative of persistent low-level air leak) and 30 days (late recurrence), requirement for further pleural procedures or thoracic surgery after ICD removal, and length of stay. Recurrence was measured from the point of ICD clamping (clamped arm) or ICD removal (nonclamped arm) (ie, at the time point when a decision regarding clinically relevant cessation of air leak was undertaken in both arms). Length of stay was calculated for the index admission and, where relevant, as total length of stay combining the index and subsequent readmission with recurrent pneumothorax.

Patient demographic data are presented as absolute values, percentages, and medians (interquartile range [IQR]). Statistical analyses were performed in Python (3.10.9; Python Software Foundation) using SciPy (1.11.1),^22^ and in R (4.4.3; R Foundation for Statistical Computing) using lme4 (1.1-37).^23^ Mann-Whitney *U* test was used for comparisons between continuous variables with nonnormal distribution. Log-rank test was used to compare cumulative incidence. The Pearson χ^2^ test was used for categorical variables, and if expected frequencies were low, a Fisher exact test was used instead. A generalized linear mixed model was used for adjusted analyses, including key variables (age, PSP vs SSP, prior pneumothorax, smoking status, ICD diameter, and suction) and a random intercept for sites to account for clustering. *P* < .05 was considered statistically significant.

## Results

**Figure 1:**
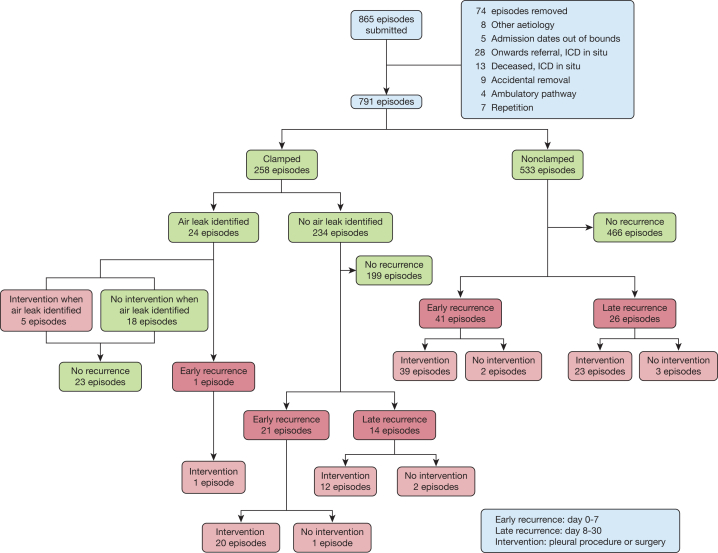
Flowchart of patients included in the Characterising Leak of Air in Medical Pneumothorax study. Early recurrence is defined as pneumothorax identified on or before day 7 from time of clamping (clamped group) or chest drain removal (nonclamped group), whereas late is from day 8 to 30. Intervention is inclusive of any attempted pleural procedure or surgery for pneumothorax. ICD = intercostal chest drain.

A total of 791 admissions with pneumothorax from 27 UK hospitals were included in the analysis (Fig 1, Table 1, e-Fig 1). The median age of patients in the study was 54 years (IQR, 24-70), with a bimodal peak of younger patients with PSP (median age, 31 years; IQR, 26-39) and older patients with SSP (median age, 66; IQR, 54-75) (e-Table 2). Most patients included presented with SSP (63.5%, 502 of 791), for which the most commonly identified etiologies were COPD (60.8% of SSP, 305 of 502) and presumed COPD (16.1%, 82 of 502).^2^ For most patients, this admission represented their first pneumothorax (80.4%, 636 of 791).

**Table 1:** Demographic and Clinical Details Separated by Clamping Group vs Nonclamped Group

Demographic	Total (N = 791)	Clamp (n = 258)	No Clamp (n = 533)
Age, y	54 (34.0-70.0)	53 (33.3-67.0)	55 (34.0-72.0)
Region			
North East	171 (21.6)	83 (32.2)	88 (16.5)
Yorkshire and the Humber	132 (16.7)	6 (2.3)	126 (23.6)
East of England	106 (13.4)	42 (16.3)	64 (12.0)
West Midlands	94 (11.9)	17 (6.6)	77 (14.4)
Kent, Surrey, and Sussex	57 (7.2)	17 (6.6)	40 (7.5)
South West (Peninsula region)	54 (6.8)	22 (8.5)	32 (6.0)
Thames Valley	53 (6.7)	29 (11.2)	24 (4.5)
Northern Ireland	33 (4.2)	3 (1.2)	30 (5.6)
North West	25 (3.2)	10 (3.9)	15 (2.8)
East Midlands	23 (2.9)	9 (3.5)	14 (2.6)
London	17 (2.1)	9 (3.5)	8 (1.5)
South West (Severn region)	16 (2.0)	5 (1.9)	11 (2.1)
Wales	10 (1.3)	6 (2.3)	4 (0.8)
PSP vs SSP			
PSP	289 (36.5)	93 (36.0)	196 (36.8)
SSP	502 (63.5)	165 (64.0)	337 (63.2)
SSP, etiology			
COPD	305 (60.8)	105 (63.6)	200 (59.3)
Presumed COPD	81 (16.1)	16 (9.7)	65 (19.3)
Asthma	27 (5.4)	5 (3.0)	22 (6.5)
ILD	24 (4.8)	12 (7.3)	12 (3.6)
Infection	16 (3.2)	8 (4.8)	8 (2.4)
Cystic lung disease	8 (1.6)	2 (1.2)	6 (1.8)
Malignancy	15 (3.0)	6 (3.6)	9 (2.7)
Other	26 (5.2)	11 (6.7)	15 (4.5)
Smoking status			
Current	321 (40.6)	98 (38.0)	223 (41.9)
Ex	269 (34.1)	90 (34.9)	179 (33.6)
Never	167 (21.1)	58 (22.5)	109 (20.5)
Unknown	33 (4.2)	12 (4.7)	21 (3.9)
Pneumothorax laterality			
Right	446 (56.4)	149 (57.8)	297 (55.7)
Left	345 (43.6)	109 (42.2)	236 (44.3)
Prior pneumothorax			
No	636 (80.4)	208 (80.6)	428 (80.3)
Yes (ipsilateral)	108 (13.7)	40 (15.5)	68 (12.8)
Yes (contralateral)	22 (2.8)	8 (3.1)	14 (2.6)
Yes (unknown)	25 (3.2)	2 (0.8)	23 (4.3)
Chest drain diameter, French gauge			
< 12	7 (0.9)	3 (1.2)	4 (0.8)
12	575 (72.3)	196 (76.0)	379 (71.1)
14-18	94 (11.9)	28 (10.9)	66 (12.4)
> 18	15 (1.9)	6 (2.3)	9 (1.7)
Unknown	100 (12.6)	25 (9.7)	75 (14.1)
Suction			
Wall	187 (23.6)	75 (29.1)	112 (21.0)
Digital	58 (7.3)	24 (9.3)	34 (6.4)
No suction	546 (69.0)	159 (61.6)	387 (72.6)

Data are presented as median (interquartile range) or No. (%). ILD = interstitial lung disease; PSP = primary spontaneous pneumothorax; SSP = secondary spontaneous pneumothorax.

**Figure 2:**
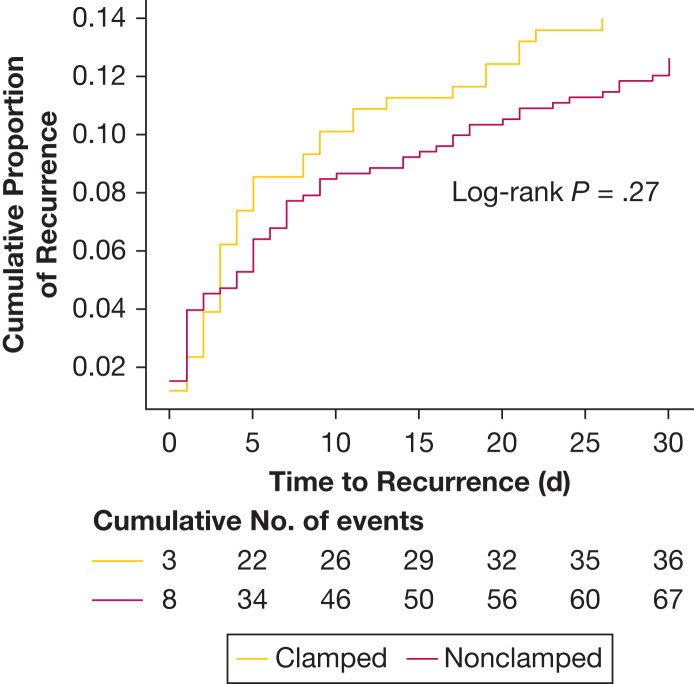
Pneumothorax recurrence within 30 d of chest drain removal. Cumulative events curve from time of chest drain removal separated by those in clamped group or nonclamped group. Log-rank test demonstrated no significant difference between the 2 curves (*P* = .27). Pearson χ^2^ test demonstrated no significant difference in recurrence at day 7 (*P* = .79).

Overall, 13.0% of patients (103 of 791) had recurrence of pneumothorax within 30 days. Early recurrence (within 7 days) occurred in 8.0% (63 of 791) of total cases. Clamping trials were undertaken in 32.6% of episodes (258 of 791) (Fig 1). The demographics were similar for the subgroups for patients who did or did not have a trial of clamping, with no difference in clamping frequency identified between patients with PSP and SSP (clamping performed in 32.2% vs 32.9% of episodes) (Table 1). Persistent air leak was identified during clamping trials by performing a chest radiograph, with a median time after clamping of 240 minutes (IQR, 180-420). A clamping trial was found to be positive (ie, revealed an ongoing air leak, resulting in unclamping of the drain) in 9.3% of episodes (24 of 258): 5 of these patients were managed with a second ICD insertion, and 11 underwent a further clamping trial, of which 1 was discharged and underwent additional pleural intervention for a later recurrence. Thus, a maximum of 18 patients avoided a further pleural procedure (Fig 1). Where a clamping trial revealed no ongoing air leak, 35 of 234 patients (15.0%) were ultimately diagnosed with recurrent pneumothorax, of which most (21 of 35) were diagnosed within the first 7 days from the trial. Of these 35 patients, 32 required an intervention, including 20 of the 21 early recurrences. In the nonclamped group, 7.7% (41 of 533) had early recurrence, with 39 (7.3%) requiring a further pleural procedure, of which 16 took place during the index admission. There was no significant difference in early recurrence of pneumothorax between cases where a clamping trial had or had not been undertaken (8.5% clamped vs 7.7% nonclamped; *P* = .07, χ^2^) (Fig 1, Table 2). Adjusted analyses accounting for key confounders and clustering between sites also showed no significant difference in early recurrence. Rates of total recurrence within 30 days, late recurrence alone (day 8-30), and time to recurrence were also similar between clamped and nonclamped arms. (Fig 2).

**Table 2:** Results Comparing Outcomes Between Clamped Group vs Nonclamped Group

Outcome	Total (N = 791)	Clamp (n = 258)	No Clamp (n = 533)	*P* Value
Recurrence ≤ 7 d	63 (8.0)	22 (8.5)	41 (7.7)	.79
Proportion PSP	20 (31.7)	6 (27.3)	14 (34.1)	.78
Proportion SSP	43 (68.3)	16 (72.7)	27 (65.9)	.78
Proportion of people who currently smoke	17 (27.0)	2 (9.1)	15 (36.6)	.04
Proportion requiring pleural intervention	60 (95.2)	21 (95.5)	39 (95.1)	> .99
Excluding suction (n = 546)	40/546 (7.3)	14/159 (8.8)	26/387 (6.7)	.50
Recurrence ≤ 30 d	103 (13.0)	36 (14.0)	67 (12.6)	.67
Proportion PSP	31 (30.1)	10 (27.8)	21 (31.3)	.88
Proportion SSP	72 (69.9)	26 (72.2)	46 (68.7)	.88
Proportion of people who currently smoke	35 (34.0)	7 (19.4)	28 (41.8)	.04
Proportion requiring pleural intervention	95 (92.2)	33 (91.7)	62 (92.5)	> .99
Excluding suction (n = 546)	69/546 (12.6)	23/159 (18.7)	46/387 (11.9)	.50
Length of stay, d	5 (3.0-9.0)	6 (3.0-11.0)	5.0 (3.0-9.0)	.08^a^
Length of time chest drain in situ, d	4 (2.0-6.0)	4 (2.0-8.0)	3 (2.0-6.0)	.0002^a^

Data are presented as median (interquartile range), No. (%), No./total No. (%), or as otherwise indicated. PSP = primary spontaneous pneumothorax; SSP = secondary spontaneous pneumothorax.

a Mann-Whitney *U* test.

Adverse events associated with clamping occurred in 2.3% of patients (n = 6) and included symptoms in keeping with persistent air leak (pain and breathlessness), 1 case of subcutaneous emphysema, and 1 case consistent with a diagnosis of tension pneumothorax (hypotension alongside symptoms in keeping with persistent breathlessness). No association was identified between the length of clamping trial and the rate of early recurrence (*P* = .99, Mann-Whitney *U* test). Use of suction either alongside clinical assessment of air leak via an underwater seal or via a digital air leak device was more frequent in the clamped group, with 38.4% (99 of 258) having had suction applied compared with 27.4% (146 of 533) in the nonclamped category. Median length of stay during the index admission was 6 days (IQR, 3-11) in patients with clamping trials and 5 days (IQR, 3-9) in those without (*P* = .08, Fisher exact test). Median total length of stay (including the index episode and subsequent readmissions) was also 6 days in the clamped arm and 5 days in the nonclamped arm.

Rates of pneumothorax recurrence were not significantly different at either early or late time points when digital air leak devices were used, compared with patients who did not undergo suction and those who were treated via traditional suction methods (*P* = .24 and *P* = .59, χ^2^) (Table 3, e-Table 3). When digital air leak devices and clamping were used in combination (n = 24), rates of recurrent pneumothorax were the lowest within this data set, with no cases (0.0%) within 7 days of clamping and 1 case (4.2%) within 30 days; however, given the small number of patients treated with this approach, these differences did not reach statistical significance (*P* = .17 and *P* = .29, Fisher exact test).

**Table 3:** Results Comparing Outcomes Between Digital Suction vs Other Groups

Suction (With or Without Clamping)	Digital Suction (n = 58)	Wall Suction + No Suction (n = 733)	*P* Value
Recurrence ≤ 7 d	3 (5.2)	60 (8.2)	.61^a^
Recurrence ≤ 30 d	6 (10.3)	97 (13.2)	.67

	Digital Suction (n = 58)	Wall Suction (n = 187)	No Suction (n = 546)	*P* Value
Recurrence ≤ 7 d	3 (5.2)	20 (10.7)	40 (7.3)	.24
Recurrence ≤ 30 d	6 (10.3)	28 (15)	69 (12.6)	.59

Data are presented as No. (%) or as otherwise indicated.

a Fisher exact test.

## Discussion

To our knowledge, we report the largest study undertaken of ICD removal strategies. We found recurrent air leak after ICD removal to be common, complicating 8.0% of cases by day 7, higher than has been previously published.^2^ Recurrence of pneumothorax by day 30, representing a composite of failed healing of the initial defect and interval development of a second puncture, occurred in 13.0% of cases, emphasizing that this is a group of patients at high risk of respiratory deterioration after apparent completion of treatment. All current strategies for assessing the risk of recurrence were found to be inadequate, unable to reliably exclude ongoing low volume air leak. In particular, clamping trials were not associated with reduced rates of pneumothorax recurrence when compared with usual care of monitoring of air leak via underwater seal or digital air leak devices (nonclamped arm). This could relate to persistent air leak being too low to drive lung collapse and respiratory symptoms during short clamping trials only becoming apparent at later time points. Positive clamping trials may have saved a maximum of 18 additional pleural procedures in patients who did not require further intervention (7.0% [18 of 258] of the clamped arm). However, 15% of patients who had a negative clamping trial still developed early recurrence.

Because criteria determining ICD removal strategies were not set, patients who underwent clamping could represent a subgroup thought by clinicians to be at high risk of recurrent pneumothorax. Although the most obvious confounders (SSP and previous pneumothorax) were not different between the clamped and nonclamped groups (Table 1), this observational study remains at risk of confounding from other unmeasured variables. Similarly, data concerning suction must be interpreted with caution because it is likely that particular subgroups had different likelihoods of receiving this treatment, depending on the nature of their air leak (eg, slow to resolve) or their underlying pathology. The role of suction in spontaneous pneumothorax remains unclear with prospective trials ongoing.^24^ Air reaccumulation after clamping in this study was identified by a single chest radiograph: others have recommended serial radiographs to assess more sensitively for ongoing pressure-dependent air leak; however, the most common causes of pressure-dependent pneumothorax (eg, postsurgical) were excluded from this study.^25^ Although the increased length of stay (6 vs 5 days) in patients who underwent clamping trials was not statistically significant, the study was not specifically designed to answer this question, and this signal should be an important focus of any future prospective work.

Although clamping trials were generally safe, to our knowledge, this study did identify the first clearcut case of tension pneumothorax secondary to ICD clamping described in the medical literature. In 2 previous studies of ICD removal, tension hemodynamics postclamping were identified in 0 of 214 trauma patients by Becker at al^12^ (although 1 patient did develop hypoxemia) and in 0 of 68 patients with spontaneous pneumothorax by Chan et al.^11^ Funk et al^26^ identified 1 (of 134) patient requiring urgent second ICD insertion after clamping in trauma; however, this was thought to be due to tube occlusion by thrombus, presumably in the context of hemothorax, and more detailed data on complications were not included.^26^ Our study is therefore unique in being able to estimate the overall rate of tension pneumothorax precipitated by chest drain clamping as 0.4%. It is important to consider, however, that ICD removal for this patient may have resulted in the same physiological decompensation as clamping. It can therefore be argued that detecting this before ICD removal permits more straightforward resolution, by unclamping the ICD rather than necessitating repeat pleural intervention.

Before this study, evidence regarding ICD clamping in spontaneous pneumothorax was limited. One single-center retrospective analysis of 122 patients with spontaneous pneumothorax found that clamping trials were successful in detecting pneumothorax recurrence within 24 hours in 17.4% of patients (n = 12) compared with 7.4% (n = 4) in the nonclamped group.^11^ The authors suggest that clamping may have prevented ICD reinsertion in 11.8% of cases (n = 8).^11^ One prospective, randomized study of clamping (including few patients with spontaneous pneumothorax) identified that ICD removal guided by digital drainage systems alone was noninferior to the use of clamping trials for a primary outcome of ICD reinsertion within 24 hours.^13^ ICD clamping has also been assessed in trauma, where a prospective trial has been conducted and did not find any difference in the rate of recurrent pneumothorax with clamping.^14^ The largest previous study of clamping was a retrospective review of 499 patients with hemothorax and pneumothorax, which found that the need for ipsilateral pleural drainage within 30 days was 6% (n = 13) when ICDs were clamped compared with 12% (n = 33) in the nonclamped arm.^12^ One further smaller retrospective study in trauma concluded that clamping may have utility in detecting recurrence; however, requirement for ICD reinsertion was actually higher in the clamped group.^26^

The utility of digital air leak devices postsurgery has not yet been extended to spontaneous pneumothorax, with 1 randomized controlled trial of digital air leak devices in PSP showing no significant difference in ICD duration, length of stay, or rate of recurrent pneumothorax between the 2 arms.^10^^,^^27^ In our study, an ultracautious approach of using both digital air leak measurement and clamping was associated with the lowest rates of pneumothorax recurrence, and this may therefore constitute a promising strategy in selected cases (Table 3). The efficacy of digital air leak devices is difficult to disentangle because they both provide suction and allow objective measurement of air leak: in the context of ICD removal, it is likely that the latter is the dominant driver of utility.

Major strengths of this study include the size of the cohort assessed, which represents the largest study of ICD removal yet published. In addition to the large number of patients included, it was conducted in 27 centers across the United Kingdom (previous studies have been single center), making our results more robust. This is essential for the generation of relevant data, given the substantial variability in physician practice.^16^^,^^17^ Therefore, although individual patient factors should always be taken into consideration, the clarity on the typical expected clinical course for a patient having an ICD removed, with or without a preceding clamping trial, provided by this study is likely to be helpful for physicians and patients making this shared decision.

The primary limitation of this study is that it was a retrospective case series and therefore cannot be considered evidence of any causal link between ICD removal strategies and the outcomes measured. This approach was intentional and is justified because high-quality observational data are required before undertaking a prospective randomized controlled trial. As discussed previously, criteria determining ICD removal strategy were not set; therefore, the study is limited by the risk of selection bias, which would again need to be addressed by a prospective randomized controlled trial. A primary hypothesis was also not prespecified; therefore, all subgroup analyses were exploratory. The lack of collection of certain demographic details (ie, sex) could limit generalizability; however, the broad inclusion criteria, multicenter approach, and consistency of other details (eg, age) with known patterns of pneumothorax presentation suggests this population is representative. The data set does not include patients with a continued large air leak for whom clamping trials would not be recommended. The decision to omit patients who had undergone pleurodesis from the data set could have excluded certain patients thought to be at high risk for recurrence. Finally, in the UK setting, definitive surgical intervention for pneumothorax may be undertaken at a different hospital to the index presentation; thus, it can be challenging to collect accurate data on total length of admission for patients who required onward referral.

## Interpretation

This study shows that pneumothorax recurrence after ICD removal is a common complication. Clamping trials are safe, but do not reduce the rate of recurrent pneumothorax or need for repeat pleural intervention. How decisions are made regarding ICD removal remains an unanswered question and 1 which is of importance to patients, clinicians, and health care commissioners. A prospective randomized trial of ICD removal strategies including clamping and digital air leak devices is now warranted.

## Funding/Support

The study was supported by 10.13039/501100000303INSPIRE - the UK’s research network for early career respiratory clinicians (https://www.inspirerespiratory.co.uk/). Study data were collected and managed using REDCap tools [https://doi.org/10.1016/j.jbi.2019.103208] hosted within the Cambridge Integrated Data Environment (CAM:IDE), which is supported by the NIHR Cambridge Biomedical Research Centre and the University of Cambridge, Department of Psychiatry.

## Financial/Nonfinancial Disclosures

None declared.

## Supplementary Data

e-Online Data

e-Online Data
