# Supplementary material for: A National Evaluation of Intercostal Chest Drain Removal Strategies
Source: Chest. 2025 Nov 4;169(3):849–58. doi: 10.1016/j.chest.2025.10.027 (PMC12975392; doi:10.1016/j.chest.2025.10.027)

**Supplementary legends**

**Supplementary Table 1:** Completion counts for key analytic variables used in complete case analysis.

**Supplementary Table 2**: Demographic and clinical details separated by PSP or SSP. IQR, interquartile range.

**Supplementary Table 3:** Expanded results comparing outcomes of use of suction separated by those who are also clamped group versus non-clamped group.

* Fisher’s exact test

**Supplementary Figure 1:** **Distribution of NHS trusts contributing cases retrospectively to CLAMP study.** Labelled are the 10 highest contributing trusts. Legend indicative of the number of cases contributed.

**Supplementary Table 1**

| **Category** | **Variable** | **Number of records complete (%)** |
| --- | --- | --- |
| **Demographic** | NHS Trust | 791/791 (100.0%) |
|  | Date of admission | 791/791 (100.0%) |
|  | Age | 791/791 (100.0%) |
|  | PSP vs SSP | 791/791 (100.0%) |
|  | SSP aetiology | 500/502 (99.6%) |
|  | Pneumothorax laterality | 791/791 (100.0%) |
|  | Prior pneumothorax | 791/791 (100.0%) |
|  | Smoking status | 757/791 (95.7%) |
|  | ICD diameter | 691/791 (87.4%) |
| **Suction** | Suction used | 791/791 (100.0%) |
| **Clamping** | Clamp | 791/791 (100.0%) |
|  | Interval between clamp and CXR | 250/258 (96.9%) |
|  | CXR result | 258/258 (100.0%) |
|  | Adverse events from clamping | 258/258 (100.0%) |
| **Discharge** | Date of ICD removal | 791/791 (100.0%) |
|  | Date of discharge | 791/791 (100.0%) |
|  | Length of stay | 791/791 (100.0%) |
|  | Length of time ICD in situ | 791/791 (100.0%) |
| **Recurrence** | Recurrence | 791/791 (100.0%) |
|  | Date of recurrence | 103/103 (100.0%) |
|  | Interval between ICD removal to recurrence | 103/103 (100.0%) |
|  | Recurrence management | 103/103 (100.0%) |
|  | Intervention required | 103/103 (100.0%) |
|  | Date of discharge on recurrence admission | 48/78 (61.5%) |

**Supplementary Table 2**

| **Demographics (PSP vs SSP)** | | | |
| --- | --- | --- | --- |
|  | | **PSP**  **(N=289)** | **SSP**  **(N=502)** |
| **Median age (IQR), years** |  | 31 (26.0-39.0) | 66 (54.0-75.0) |
| **Smoking status** |  | | |
|  | Current | 129 (44.6%) | 192 (38.3%) |
|  | Ex | 40 (13.8%) | 229 (45.7%) |
|  | Never | 108 (37.4%) | 59 (11.8%) |
|  | Unknown | 12 (4.2%) | 21 (4.2%) |
| **Pneumothorax laterality** |  | | |
|  | Right | 168 (58.1%) | 278 (55.4%) |
|  | Left | 121 (41.9%) | 224 (44.6%) |
| **Prior pneumothorax** |  | | |
|  | No | 232 (80.3%) | 404 (80.5%) |
|  | Yes (ipsilateral) | 44 (15.2%) | 64 (12.7%) |
|  | Yes (contralateral) | 5 (1.7%) | 17 (3.4%) |
|  | Yes (unknown) | 8 (2.8%) | 17 (3.4%) |
| **ICD diameter,**  **French gauge** |  | | |
|  | <12 | 1 (0.3%) | 6 (1.2%) |
|  | 12 | 211 (73.0%) | 364 (72.5%) |
|  | 14-18 | 36 (12.5%) | 58 (11.6%) |
|  | >18 | 5 (1.7%) | 10 (2.0%) |
|  | Unknown | 36 (12.5%) | 64 (12.7%) |
| **Clamping trials** |  | | |
|  | Clamping | 93 (32.2%) | 165 (32.9%) |
|  | No clamping | 196 (67.8%) | 337 (67.1%) |
| **Suction** |  | | |
|  | Nil | 174 (60.2%) | 372 (74.1%) |
|  | Wall | 91 (31.5%) | 96 (19.1%) |
|  | Digital | 24 (8.3%) | 34 (6.8%) |
| **Recurrence** |  | | |
|  | Recurrence ≤ 7 days | 20 (6.9%) | 43 (8.6%) |
|  | Recurrence ≤ 30 days | 31 (10.7%) | 72 (14.3%) |
| **Length of stay (IQR), days** | | 4 (3.0-6.0) | 6 (3.0-12.0) |
| **Length of time ICD in situ (IQR), days** | | 2 (2.0-6.0) | 4 (2.0-7.0) |

**Supplementary Table 3**

| **≤Suction without clamping (N=533)** | | | |
| --- | --- | --- | --- |
|  | **All suction**  **(N=146)** | **No suction**  **(N=387)** |  |
| **Recurrence ≤ 7 days – No. (%)** | 15 (10.3%) | 26 (6.7%) | *P*=0.23 |
| **Recurrence ≤ 30 days – No. (%)** | 21 (14.4%) | 46 (11.9%) | *P*=0.53 |
|  | | | |
|  | **Wall suction**  **(N=112)** | **No suction**  **(N=387)** |  |
| **Recurrence ≤ 7 days – No. (%)** | 12 (10.7%) | 26 (6.7%) | *P*=0.23 |
| **Recurrence ≤ 30 days – No. (%)** | 16 (14.3%) | 46 (11.9%) | *P*=0.61 |
|  |  |  |  |
|  | **Digital suction**  **(N=34)** | **No suction**  **(N=387)** |  |
| **Recurrence ≤ 7 days – No. (%)** | 3 (8.8%) | 26 (6.7%) | *P*=0.72* |
| **Recurrence ≤ 30 days – No. (%)** | 5 (14.7%) | 46 (11.9%) | *P*=0.59* |
|  | | | |
|  | **Digital suction**  **(N=34)** | **Wall suction**  **(N=112)** |  |
| **Recurrence ≤ 7 days – No. (%)** | 3 (8.8%) | 12 (10.7%) | *P*=1.0* |
|  | | | |
| **Suction with clamping (N=258)** | | | |
|  | **All suction**  **(N=99)** | **No suction**  **(N=159)** |  |
| **Recurrence ≤ 7 days – No. (%)** | 8 (8.1%) | 14 (8.8%) | *P*=1.0 |
| **Recurrence ≤ 30 days – No. (%)** | 13 (13.1%) | 23 (14.5%) | *P*=0.91 |
|  | | | |
|  | **Wall suction**  **(N=75)** | **No suction**  **(N=159)** |  |
| **Recurrence ≤ 7 days – No. (%)** | 8 (10.7%) | 14 (8.8%) | *P*=0.83 |
| **Recurrence ≤ 30 days – No. (%)** | 12 (16.0%) | 23 (14.5%) | *P*=0.91 |
|  | | | |
|  | **Digital suction**  **(N=24)** | **No suction**  **(N=159)** |  |
| **Recurrence ≤ 7 days – No. (%)** | 0 (0.0%) | 14 (8.8%) | *P*=0.17* |
| **Recurrence ≤ 30 days – No. (%)** | 1 (4.2%) | 23 (14.5%) | *P*=0.29* |
|  | | | |
|  | **Digital suction**  **(N=24)** | **Wall suction**  **(N=75)** |  |
| **Recurrence ≤ 7 days – No. (%)** | 0 (0.0%) | 8 (10.7%) | *P*=0.19* |

**Supplementary Fig. 1**


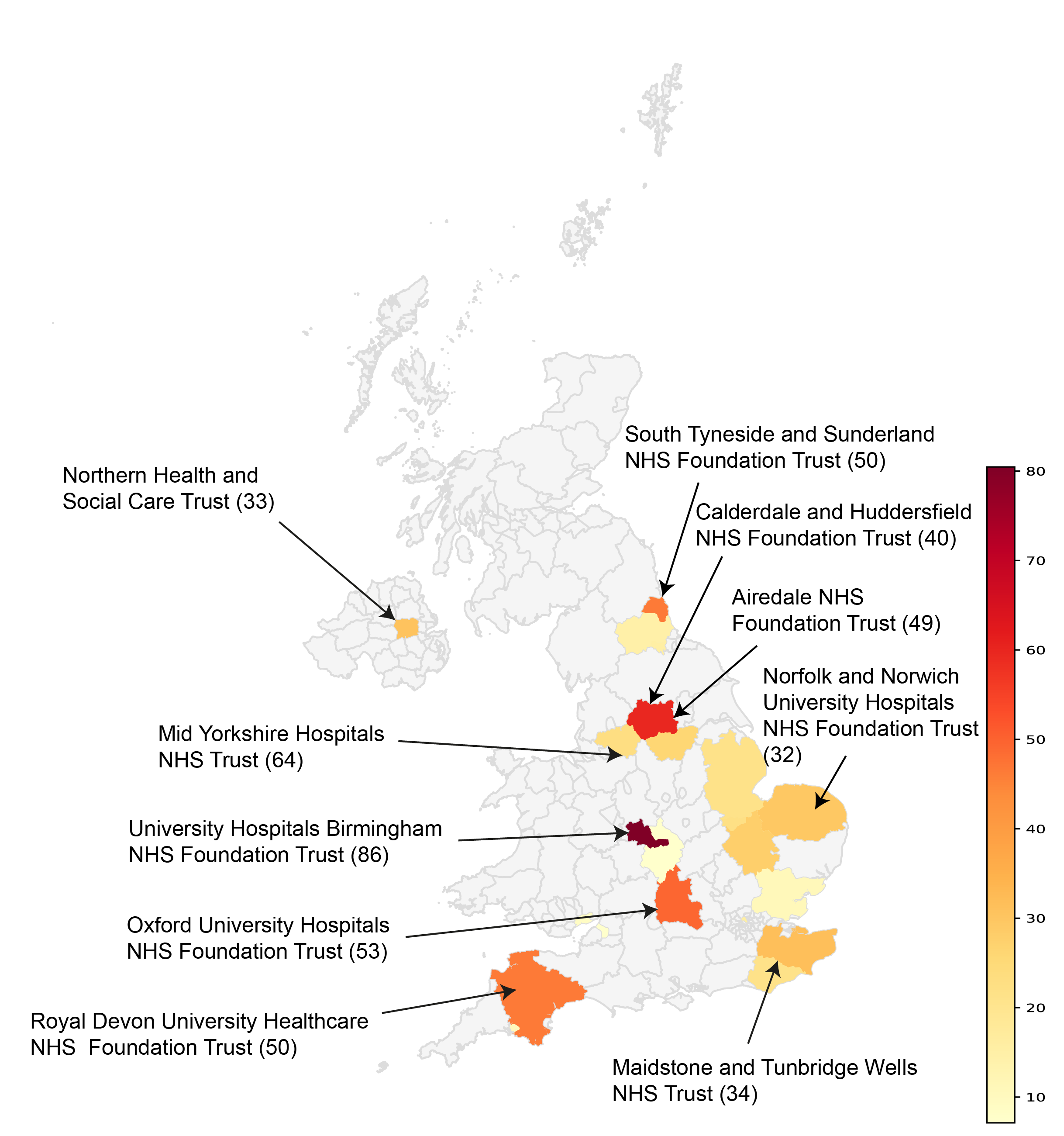

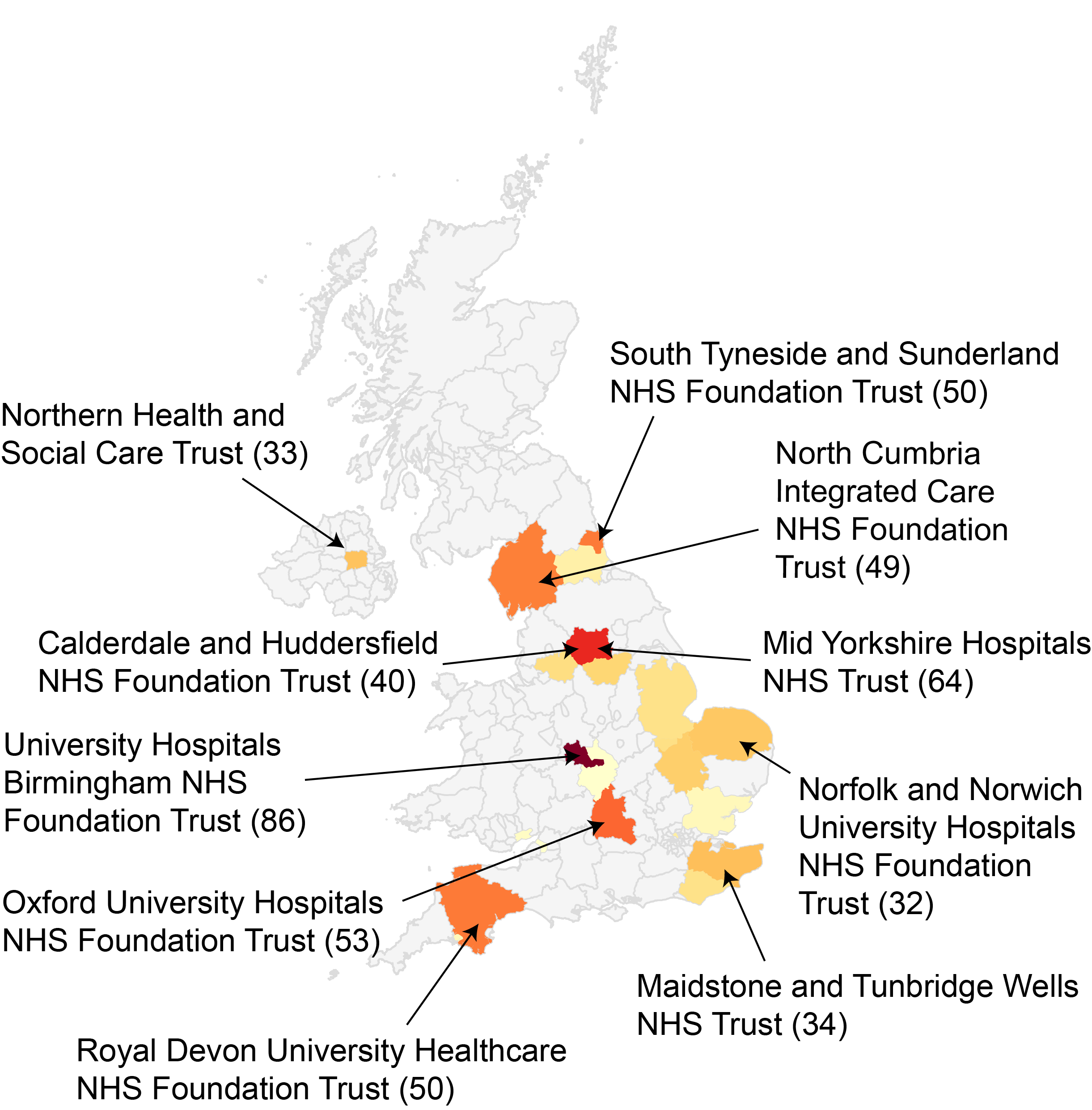

Supplement: e-Online Data [file mmc1.docx]
